# Anterior Superior Longitudinal Fasciculus Microstructure Correlates With Bimanual Visuomotor Performance in Healthy Younger but Not Older Adults

**DOI:** 10.1111/ejn.70619

**Published:** 2026-07-14

**Authors:** Mikael Novén, Jesper Lundbye‐Jensen, Henrik Lundell, Hartwig Roman Siebner, Anke Ninija Karabanov

**Affiliations:** ^1^ Section for Movement and Neuroscience, Department of Nutrition, Exercise and Sports University of Copenhagen Copenhagen Denmark; ^2^ Danish Research Centre for Magnetic Resonance, Department of Radiology and Nuclear Medicine Copenhagen University Hospital, Amager and Hvidovre Hvidovre Denmark; ^3^ Department of Health Technology Technical University of Denmark Lundtofte Denmark; ^4^ Department of Neurology Copenhagen University Hospital Bispebjerg and Frederiksberg Copenhagen Denmark; ^5^ Institute for Clinical Medicine, Faculty of Medical and Health Sciences University of Copenhagen Copenhagen Denmark

**Keywords:** bimanual coordination, brain–behavior relationships, cortical thickness, healthy aging, white matter microstructure

## Abstract

Bimanual motor performance declines with age and is accompanied by cortical thinning and alterations in white matter microstructure within motor control networks. Aging is also associated with increased interindividual variability in behavioral and structural markers, and some studies report stronger brain–behavior associations in older compared with younger adults. We tested whether bimanual performance is related differently to structural brain properties in younger and older adults. Twenty‐three younger (22–27 years) and 23 older adults (65–70 years) performed a visually guided bimanual pinch‐force task and underwent whole‐brain structural magnetic resonance imaging (MRI). Fractional anisotropy (FA) and cortical thickness (CTh) were extracted from a predefined bilateral visuomotor network and compared between groups. In regions showing significant age‐related differences, we tested whether and how FA and CTh values correlate with bimanual performance within each age group. The older age group showed lower FA in the anterior portions of the bilateral superior longitudinal fasciculus (SLF) III and right inferior fronto‐occipital fasciculus (IFOF) and bilaterally in the corpus callosum (CC) tract connecting the supplementary motor area (SMA) of each hemisphere. They also showed lower CTh across most visuomotor regions, along with lower bimanual performance compared with the younger group. Bimanual performance correlated positively with FA in the anterior segment of the right SLF III in younger but not older adults. Furthermore, larger hemispheric asymmetries in cortical thickness between the dominant and nondominant SMA correlated positively with larger intermanual performance differences between the dominant and nondominant hand in the younger but not older age group. However, the differences between age groups in structure–performance correlations were not statistically significant. Taken together, our findings suggest that intrahemispheric white‐matter integrity and interhemispheric differences in CTh support efficient bimanual control in early adulthood, but they provide no evidence that structure–function relationships increase with age.

## Graphical abstract

Younger adults showed stronger links between bimanual visuomotor control and white‐matter integrity in frontoparietal pathways. These associations were absent in older adults, suggesting that balanced cortical structure and intact white‐matter connectivity support efficient motor coordination primarily in early adulthood.

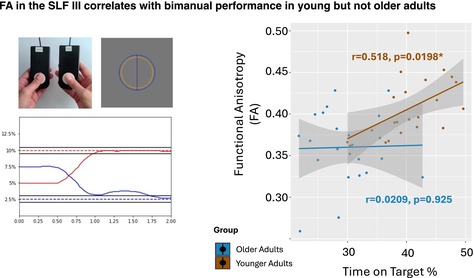

**Abbreviations**
BOLD: blood oxygen level dependent
CC: corpus callosum
CST: corticospinal tract
CTh: cortical thickness
DWI: diffusion weighted imaging
EHI: Edinburgh Handedness Inventory
EPI: echoplanar imaging
FA: fractional anisotropy
FLAIR: fluid‐attenuated inversion recovery
fMRI: functional magnetic resonance imaging
FOV: field of view
IFOF: inferior fronto‐opercular fasiculus
MD: mean diffusivity
MOCA: Montreal Cognitive Assessment
MRI: magnetic resonance imaging
MVC: maximum voluntary contraction
M1: primary motor cortex
PMd: dorsal premotor cortex
ROI: region of interest
SLF: superior longitudinal fasciculus
SMA: supplementary motor area
TE: echo time
TR: repetition time
TOI: tract of interest
ToT: time on target

## 1 Introduction

The ability to coordinate both hands is essential for many activities of daily living. However, bimanual coordination deteriorates across the adult lifespan (Boisgontier et al. 2018). Older adults show longer movement times, higher performance variability, and reduced movement accuracy (Kang and Cauraugh 2022; Krehbiel et al. 2017; Roman‐Liu and Tokarski 2021; Rudisch et al. 2020; Vieluf et al. 2015). This decline in bimanual function is accompanied by well‐documented age‐related changes in cortical thickness in key nodes of the bimanual visuomotor network, including the primary motor cortex (M1), premotor cortex, supplementary motor cortex (SMA), and posterior parietal cortex (Lemaitre et al. 2012), as well as degeneration of white matter microstructure of motor‐related white matter tracts such as the corticospinal tract (CST), the CC, or the SLF (Oschwald et al. 2021; Sexton et al. 2014).

Studies have shown that better bimanual performance is associated with higher callosal white matter integrity and greater gray matter volume in the cerebellum and sensorimotor areas across age groups (Cobia et al. 2024; Johansen‐Berg et al. 2007; Koppelmans et al. 2015; Serbruyns et al. 2015). However, less is known about whether aging affects the relationship between brain structure and motor performance. Longitudinal aging studies suggest that age‐related atrophy of gray and white matter is linked to declines in manual function and demonstrate that better manual dexterity in older adults is associated with less atrophy of both gray and white matter across several cortical and subcortical regions (Dougherty et al. 2024; Zivari Adab et al. 2020). Some cross‐sectional studies have directly compared the relationship between brain structure and motor performance between older and younger adults. Some suggest age‐related differences in the relationship between callosal white matter integrity and bimanual performance measures (Serbruyns et al. 2015; Fujiyama et al. 2016) and between gray matter volume in sensori‐motor areas and the cerebellum and motor performance (Boisgontier et al. 2018), with higher brain‐performance correlations in older adults. However, this literature is not uncontested; other studies have not been able to demonstrate that age is a mediating factor in brain–behavior relationships (van Ruitenbeek et al. 2016), and others found stronger correlations between motor performance measures, such as reaction time and callosal white matter integrity in younger compared with older adults (Madden et al. 2004).

A limited number of studies have included direct comparisons in structure–motor performance associations between different age groups. Data by Boisgontier and coworkers compared performance in a bimanual visuomotor task across different age groups. They found that bimanual coordination performance was predicted by gray matter volume in the cerebellum in the groups with the worst task performance: children and adolescents, and adults over 60, with the primary sensorimotor cortex additionally contributing in the older group. In contrast, young adults, who showed the least individual variability, had no significant structure–performance correlations (Boisgontier et al. 2018). Another study found stronger and more extensive correlations between various aspects of bimanual coordination and subregions of the corpus callosum in older compared with younger adults (Serbruyns et al. 2015).

Among diffusion tensor metrics, FA is considered a sensitive marker of age‐related white‐matter decline, showing robust tractwise effects (Behler et al. 2021; Matijevic and Ryan 2021) and has therefore often been chosen as the main outcome variable. Previous studies on the association between white FA and bimanual control have focused almost exclusively on transcallosal tracts, potentially overlooking other important tracts. Functional and structural MRI studies suggest that dynamic, visually guided bimanual movements rely heavily on frontoparietal connections like the SLF (Karabanov et al. 2023; Karabanov et al. 2019; Irmen et al. 2020). Older adults show performance decline in these tasks, but the age‐related differences in brain connectivity between age groups in dynamic, visually guided bimanual movements remain largely unexamined (Wulff‐Abramsson et al. 2025; Zvornik et al. 2024).

Task differences may also influence age‐dependent differences in the structure–performance relationship. Age‐related changes in the structure–performance relationships vary considerably by task type and task demands (Razlighi et al. 2017). For example, a large study (> 600 participants) found that the correlations between memory and white matter integrity in hippocampal pathways declined after 60 years, while correlations with language or fluid intelligence remained stable across the lifespan. This highlights that for some tasks, structure–performance coupling is lower in aging individuals (de Mooij et al. 2018).

This study aims to investigate the relationship between brain structure and bimanual performance, and how it differs between age groups, using an approach that does not a priori focus on the corpus callosum. Instead, the study first identifies which structures show age‐related differences in white matter microstructure (FA) and cortical thickness within the brain's bimanual control network. Second, it examines, within these regions, whether the association between structural measures and bimanual performance shows different patterns depending on the age group. Based on prior evidence of increased correlations between bimanual performance markers and callosal integrity, we expect stronger structure–performance associations in older adults.

## 2 Methods

### 2.1 Participants

Twenty‐nine older (OA; 65–70 years old) and 25 younger (YO; 22–27 years old) healthy adults with no history of neuropsychiatric disease and not taking any neuroactive medication were recruited to participate in this study. All included participants had normal cognitive function assessed by the Montreal Cognitive Assessment (MOCA) (Nasreddine et al. 2005) and were classified as right‐handed, as assessed by the Edinburgh Handedness Inventory (EHI) (Oldfield 1971). Three OA were excluded due to MOCA scores below our exclusion criteria of 25, two OA due to claustrophobia in the MRI scanner, two OA due to incidental findings, and two YA due to faulty scans. The final sample was thus 23 subjects in each age group. All participants gave written informed consent before participation and were reimbursed for their participation. The study was approved by the Regional Committee on Health Research Ethics of the Capital Region in Denmark (De Videnskabsetiske Komitéer‐Region H; Journal‐nr: H‐21025010).

### 2.2 Experimental Procedure

The experiment was split between 2 days of testing. On day one, participants were informed about the details of the study, gave their informed consent to participate, and completed several screening tests. On the second day, participants were welcomed to the scanning facility, underwent MRI safety screening, were instructed on the bimanual task and scanning procedure, before finally undergoing MRI scanning.

After signing consent, all participants performed a selection of motor‐cognitive background tests to verify that cognitive and motor functioning fell within an expected functional range and to identify potential group differences in mental and physical functioning. Participants were screened for cognitive functioning (MOCA score > 25) and right‐handedness (EHI). Grip and pinch strength, measured by hydraulic gauges (Baseline Lite Hydraulic hand dynamometer Prod. Nr: 12‐0241 and Baseline Lite Hydraulic Pinch Gauge Prod. Nr: 12‐0226, Elmsford, NY, USA), were used to aid calibration of the pinch force task and to quantify upper limb strength. Participants were also screened for their general physical fitness to ensure homogeneous physical activity across age groups. Aerobic fitness was assessed based on VO_2_‐max estimations from seismocardiographic recordings using Seismofit (VentriJect 2022, 2900 Hellerup, Denmark). Subjects also completed a 30‐s sit‐to‐stand test (Lein et al. 2022) and completed the International Physical Activity Questionnaire (IPAQ) (Craig et al. 2003). Bimanual dexterity was assessed using the Purdue Pegboard test (Tiffin and Asher 1948). Their highest completed academic education was also recorded on an eight‐grade scale (1: 9th grade, 2: 10th grade, 3: vocational training, 4: upper secondary school, 5: business academy, 6: bachelor's degree, 7: master's degree, and 8: PhD). Finally, a subset of the Cambridge Neuropsychological Testing Automated Battery was administered, specifically the paired associates learning, rapid visual information processing with three targets, and reaction time in five‐choice mode tasks (Fray et al. 1996). The most relevant characteristics from Day 1 are summarized here (Table 1), while other measures are reported elsewhere (Weakly et al. in submission). VO_2_ max estimates were within normal values for the age groups in Denmark (20–29 years old: male, 54.4 ± 8.4; female, 43.0 ± 7.7; 60–69 years old: male, 39.2 ± 6.7; female, 31.1 ± 5.1) (Loe et al. 2013).

**TABLE 1:** Demographic and background measurements for included participants. If nothing else is indicated, reported values are group averages ± standard deviation. The groups did differ significantly in VO_2_ max (*t*(42.8) = 6.42, *p* < 0.001), MOCA score (*t*(40.0) = 2.76, *p* = 0.009), pegboard assemblies (*t*(43.9) = 5.27, *p* < 0.001), and highest completed academic degree (*t*(31.58) = 2.13, *p* = 0.0413). The asterisk to the right of each measure denotes significant group differences.

	Young group (22–27 years old)	Older group (65–70 years old)
Sex (F/M)	11/12	11/12
Age in years (range)*	24.7 (22–27)	67.3 (65–70)
BMI	23.6 ± 3.6	25.4 ± 4.4
Pegboard Assemblies*	30.0 ± 5.6	21.2 ± 5.9
Max pinch force left	8.8 ± 2.4	8.0 ± 2.1
Max pinch force right	9.2 ± 2.2	8.4 ± 2.6
30s sit‐to‐stand	17.8 ± 3.6	17.2 ± 5.4
VO_2_ max estimate* (mL/kg/min)	46.9 ± 8.4 (average‐high for age group)	32.1 ± 7.1 (average‐high for age group)
MOCA score*	29.0 ± 0.9	28.1 ± 1.3
IPAQ total physical activity (MET‐min/week)	3450 ± 3010	4060 ± 4250
Educational level*	6.08 ± 0.86	5.17 ± 1.93

### 2.3 Bimanual Pinch Grasp Task

**FIGURE 1:**
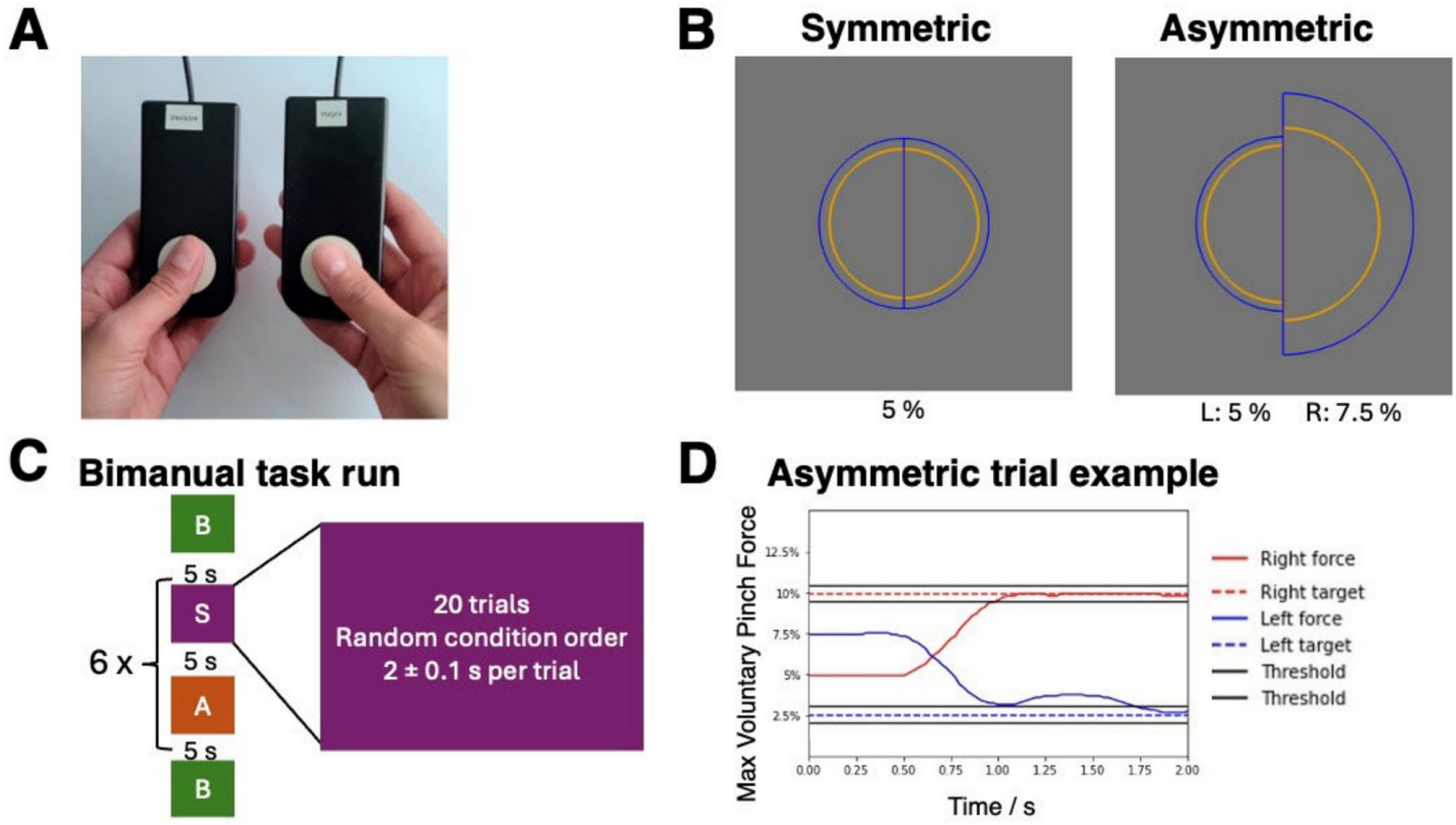
Task overview. (A) Handheld pinch force sensors are used for maximum voluntary contraction measurement and the pinch force task. (B) Example symmetric and (mirror) asymmetric trial types used in the task blocks. Pinch force applied from the left and right hands controlled the radius of the corresponding semicircle (yellow). Percentages are the target force levels (blue), relative to each subject's maximum voluntary contraction. (C) Overview of one experimental run. (D) Example performance from an asymmetric trial. Red and blue lines correspond to the left and right hands, respectively. Solid black lines indicate the accuracy threshold for the time‐on‐target measure.

After the participants underwent MRI safety screening on Day 2, participants were placed in the scanner where they were introduced to the bimanual pinch task, which consisted of dynamic transitions between different pinch force levels requiring bimanual coordination (asymmetric and symmetric blocks) and blocks of static hold at a delicate force level, requiring stable fine force control (Figure 1; see also Wulff‐Abramsson et al. 2025). When lying in the MRI scanner, participants had one force sensor, 3 cm in diameter, in a thumb pinch position in each hand (Figure 1A). Pinch force controlled the diameter of yellow semicircles on the corresponding half of a screen visible through a mirror attached to the head coil (Figure 1B). Sensor data were sampled at 1024 Hz, but the displayed force trace was smoothed, that is, averaged over the last 100 samples, to minimize electrical noise. Before the task started, the subject's max voluntary contraction (MVC) was measured for each hand, and the hand‐averaged MVC was used as a reference value for the target force levels of both hands. The session included four runs built up by a static baseline hold condition at 5% MVC for 20 s, followed by six symmetric and six asymmetric condition blocks in an interleaved order before the run was ended by another baseline hold (Figure 1C). Each block consisted of 20 trials à 2 s (±100‐ms jitter) that directly followed each other, in which the target force for each hand was either 2.5%, 5%, 7.5%, or 10% of the participant's MVC, indicated by a blue target semicircle. Each block was separated by a three‐second pause. Participants were allowed to rest between each run. Participants performed the task during four functional echo planar imaging sequences used for fMRI. While the fMRI data is reported elsewhere (Weakly et al., submitted), we use the behavioral data acquired during these scans for performance correlations with structural markers.

### 2.4 Extraction of Behavioral Measures

First, the force data were log‐transformed to comply with Weber's law of perception of change (Fechner 1860). To effectively capture bimanual performance, the time on target (ToT) was calculated as the percentage of trial time spent within 0.5% MVC of the target line (Figure 1D). ToT is sensitive to both speed and accuracy and is thus the primary measure of interest for dynamic task conditions that require subjects to reach the target as quickly as possible and maintain a stable force once the target is reached. We decided to calculate ToT as a unified behavior variable across symmetric and asymmetric blocks because previous work from our group has shown that individual performance within symmetric and asymmetric blocks is highly correlated (Weakly et al., in prep.). We also decided to average ToT across both hands, as it correlates highly between left and right hands (see Figure S1). However, as we were also interested in bimanual coordination, we additionally quantify the intermanual synchronization, and we also calculated the absolute differences between the right and left hand for ToT, as also done in our previous studies (Karabanov et al. 2023).

### 2.5 Magnetic Resonance Imaging

Data acquisition was performed at the Danish Research Centre for Magnetic Resonance, Hvidovre Hospital, Denmark, using a 3‐T MR scanner (Philips Achieva, Best, Netherlands) with a 32‐channel array receive coil. While the primary outcome measures in this study were cortical thickness and white matter microstructure, the scanning protocol also included four EPI sequences for BOLD‐fMRI. For completeness, the full acquisition protocol is described here. The scanning session started with the acquisition of a T1‐weighted image (MPRAGE; FOV: 245 mm; 245 sagittal slices, TR/TE: 6.0/2.7 ms; resolution 0.85 × 0.85 × 0.85 mm^3^; flip angle: 8°; TI: 755.9 ms) used for coregistration and quantification of cortical thickness. Four repetitions of a T2*‐weighted echo planar imaging (EPI) sequence utilizing gradient echo (FOV: 190 mm; 42 transverse slices acquired in interleaved order, TR/TE: 2000/30 ms; in‐plane resolution:3 × 3 mm^2^, 3 mm slices, no slice gap, flip angle: 90°), interleaved with four identical EPI sequences with reversed phase encoding for susceptibility distortion correction, used for functional MRI analysis (reported elsewhere: Weakly, submitted). A T2‐weighted image (TSE; FoV: 245 × 245 mm^2^; 245 sagittal slices, TR/TE: 2.5 s /270 ms; resolution 0.85 × 0.85 × 0.85 mm^3^; flip angle: 90°.; TI: 755.9 ms), a Fluid‐Attenuated Inversion Recovery image (FLAIR; 256 mm; 202 sagittal slices, TR/TE: 4.8 s/330 ms; resolution 1 × 1 × 1 mm^3^; flip angle: 90°), used for pathology assessments. Finally, one conventional diffusion‐weighted imaging (DWI) sequence (spin‐echo EPI, axial FOV: 224 × 224 mm; 66 slices; TR/TE: 7970/90; resolution: 2 × 2 × 2 mm^3^, flip angle: 90°, *b* = 0 and 2000 s/mm^2^ with 48 gradient directions) with additional 5 *b* = 0 s/mm^2^ volumes acquired using reversed phase encoding for susceptibility error correction, was acquired and used for measuring white matter microstructure parameters. One older individual could not complete the diffusion‐weighted sequence.

### 2.6 Bimanual Motor Control Network Segmentation

To define task‐relevant cortical areas and white matter tracts, we used ROIs derived from an independent dataset employing the same task (Karabanov et al. 2023). As ROI definition and hypothesis testing were conducted on nonoverlapping datasets, statistical circularity is avoided. The resulting bimanual reaching network was composed of the bilateral primary motor cortex hand area (M1hand), bilateral dorsal premotor cortex (PMd), bilateral supplementary motor area (SMA), and bilateral visual area 5, with coordinates taken from our previous work (Karabanov et al. 2023). The cortical regions of interest (ROIs) used in this study were chosen as the closest counterparts to these coordinates in Glasser's multimodal atlas (Glasser et al. 2016), except for the M1hand ROI, which was taken from another publication to focus on the hand‐knob instead of the entire M1 (Dubbioso et al. 2021). To further define the task‐relevant tracts, the TractSeg (see method below) output tracts that combined two or more of the cortical network nodes were used as tracts of interest (TOIs) for subsequent analyses. The identified TOIs were the third and fourth corpus callosal tracts (CC_3 and CC_4), the bilateral superior longitudinal fasciculus (SLF) parts I, II, and III, and the inferior fronto‐opercular fasciculus (IFOF). The CC_4 was further refined into CC M1 by using bilateral M1hand ROIs from (Dubbioso et al. 2021) as inclusion masks. The CC_3 was further refined into CC SMA by using the left and right SMA cortical ROIs as inclusion masks. As the tracts were already segmented using TractSeg, there were no additional constraints in terms of step size, angles, or FA in the CC tract refinements.

### 2.7 White Matter Microsctruture—Preprocessing and Analysis

The DWI data were used to segment TOIs and to quantify their microstructure. DWI data were preprocessed by registering the reversed phase encoding data to the main DWI b0 image using FSL's FLIRT tool (Jenkinson et al. 2002), denoising all DWI data using *dwidenoise* in MRTrix3 (Cordero‐Grande et al. 2019; Veraart et al. 2016), brain extraction using FSL's BET (Smith 2002), and finally susceptibility and eddy current distortion correction using FSL's TOPUP (J. L. R. Andersson et al. 2003) and eddy (J. L. Andersson and Sotiropoulos 2016). Next, we used FSL's *dtifit* to obtain whole‐brain fractional anisotropy (FA) and mean diffusivity (MD) maps. The maps capture different aspects of the white‐matter microstructure. FA provides a measure of axonal structure and alignment. Thereby, it is sensitive to axonal integrity and myelination (Curran et al. 2016) and it is our primary measure for white matter microstructure. MD quantifies the average mobility of water molecules in the voxel along all directions of measurement. It is thus sensitive to the general restriction of water in the tissue. Although FA was our primary, we also analyzed MD to provide complementary information on overall tissue diffusivity. To obtain the TOIs, we used TractSeg, an openly available automatic segmentation tool based on a convolutional neural network trained on data from the Human Connectome Project (Wasserthal et al. 2018). This provided anatomically valid segmentations of the white matter tracts of interest. FA and MD were sampled along 100 segments along each TOI using TractSeg's *tractometry* function (Wasserthal et al. 2020). In case of significant group differences (see “statistical analyses” below), diffusion parameter was averaged along the cohesive group of segments of the tract that showed the age group difference. As FA was the primary measure of microstructural integrity in this study, all results related to MD can be found in the Supporting Information.

### 2.8 Cortical Thickness—Preprocessing and Analysis

Cortical surface reconstruction and volumetric segmentation were performed using the FreeSurfer image analysis suite v7.2 (B. Fischl et al. 2001; Dale et al. 1999; Fischl et al. 2004; Fischl and Dale 2000). Briefly, cortical thickness (ChT) was calculated as the distance between the pial (i.e., the border of the cerebral cortex and the cerebrospinal fluid) and white (i.e., the border between the white and gray matter) surfaces. The resulting whole‐brain CTh maps were registered to FreeSurfer common space *fsaverage* and used for group difference analyses. Moreover, the average CTh within each cortical ROI using the FreeSurfer tool *mri_segstats* was calculated for each participant for specific group difference and correlational analyses.

### 2.9 Statistical Analyses

#### 2.9.1 Behavioral Performance

Group differences in ToT and bimanual symmetry were tested using independent *t*‐tests in each of the task conditions with an alpha threshold of *p* < 0.05. Shapiro–Wilk normality tests, *t*‐tests, and correlation analyses were performed with standard commands (*shapiro.test*, *t.test*, and *cor.test*, respectively) in R version 4.1.2 (R Core Team 2021). If normality was not confirmed by *p* > 0.05 in the Shapiro–Wilk test, Mann–Whitney test (*wilcox.test*) and Spearman correlation methods were used instead of *t*‐tests and Pearson correlation tests, respectively, with standard commands in R.

#### 2.9.2 Group Differences in White Matter Microstructure and Performance–Microstructure Correlations

To identify the TOI sections that were impacted by aging, each TOI was divided into 100 segments. Diffusion parameters (i.e., FA and MD) were compared between groups for each tract segment using *t*‐tests and family‐wise error correction for multiple comparisons along the tract (alpha threshold 0.05) (Wasserthal et al. 2020; Yeatman et al. 2012). In case of significant group differences that extended at least five segments along the tract, averages of the diffusion parameter from within the significant tract segment were extracted and correlated with TOT for the significant sections separately for each group. If normality was not confirmed by *p* > 0.05 in the Shapiro–Wilk test, Spearman correlation methods were used instead of Pearson. The *p* values were Bonferroni corrected across the five segments that showed significant group differences. For significant correlations, the impact of outliers were investigated by identifying datapoints with a potential large influence on the regression, defined as a Cook's distance at or above 1 (Fox 2019). Cook's distances were calculated using the “cooks.distance” function from the “stats” R package (Fox and Weisberg 2018).

#### 2.9.3 Group Differences in Cortical Thickness and Performance–CTh Correlations

To identify the cortical areas that were impacted by aging we compared CTh between groups across the whole brain using *mri_glmfit* in FreeSurfer, including permutation test for multiple comparison correction (10^4^ permutations, *p* threshold: 0.001, cluster‐wise *p* threshold: 0.05) and threshold‐free cluster enhancement (Winkler et al. 2014). Average CTh from the bimanual control network ROIs was Pearson correlations with TOT were calculated for the significant ROIs separately for each group in R. If normality was not confirmed by *p* > 0.05 in the Shapiro–Wilk test, Spearman correlation methods were used instead of Pearson. *p* values were Bonferroni corrected across the seven ROIs that showed significant group differences. For significant correlations, the impact of outliers was investigated by identifying datapoints with a potential large influence on the regression, defined as a Cook's distance at or above 1 (Fox 2019). Cook's distances were calculated using the “cooks.distance” function from the “stats” R package (Fox and Weisberg 2018).

#### 2.9.4 Correlations Between Hand Synchrony and Hemispheric Asymmetries

To explore whether there were any correlations and hand synchrony in performance and hemispheric asymmetries in brain structure, the absolute difference in performance measures between hands was Pearson correlated in *R* with the absolute difference in brain structure measure between hemispheres. For cortical ROIs, this meant the absolute difference in average cortical thickness between the left and right hemispheric homologs. For the tracts of interest, it meant the absolute difference in average diffusion parameter between the left and right hemispheric homologs. The CC tract was split into a left and right hemispheric component and considered hemispheric homologs. If normality was not confirmed by *p* > 0.05 in the Shapiro–Wilk test, Spearman correlation methods were used instead of Pearson. The *p* values were Bonferroni corrected across the four left–right ROIs where at least one hemisphere has shown significant group differences. For significant correlations, the impact of outliers was investigated by identifying datapoints with a potential large influence on the regression, defined as a Cook's distance at or above 1 (Fox 2019). Cook's distances were calculated using the “cooks.distance” function from the “stats” R package (Fox and Weisberg 2018).

#### 2.9.5 Tests for Differences in Correlation

If performance and brain structure measures correlated for any of the groups, the “cocor” R package was used to test whether the correlations within each group were significantly different, based on the computation of confidence intervals (Diedenhofen and Musch 2015). All correlation difference tests were two‐tailed, and the alpha threshold was set to 0.05 and the confidence level to 95%.

## 3 Results

### 3.1 Bimanual Task Performance

**FIGURE 2:**
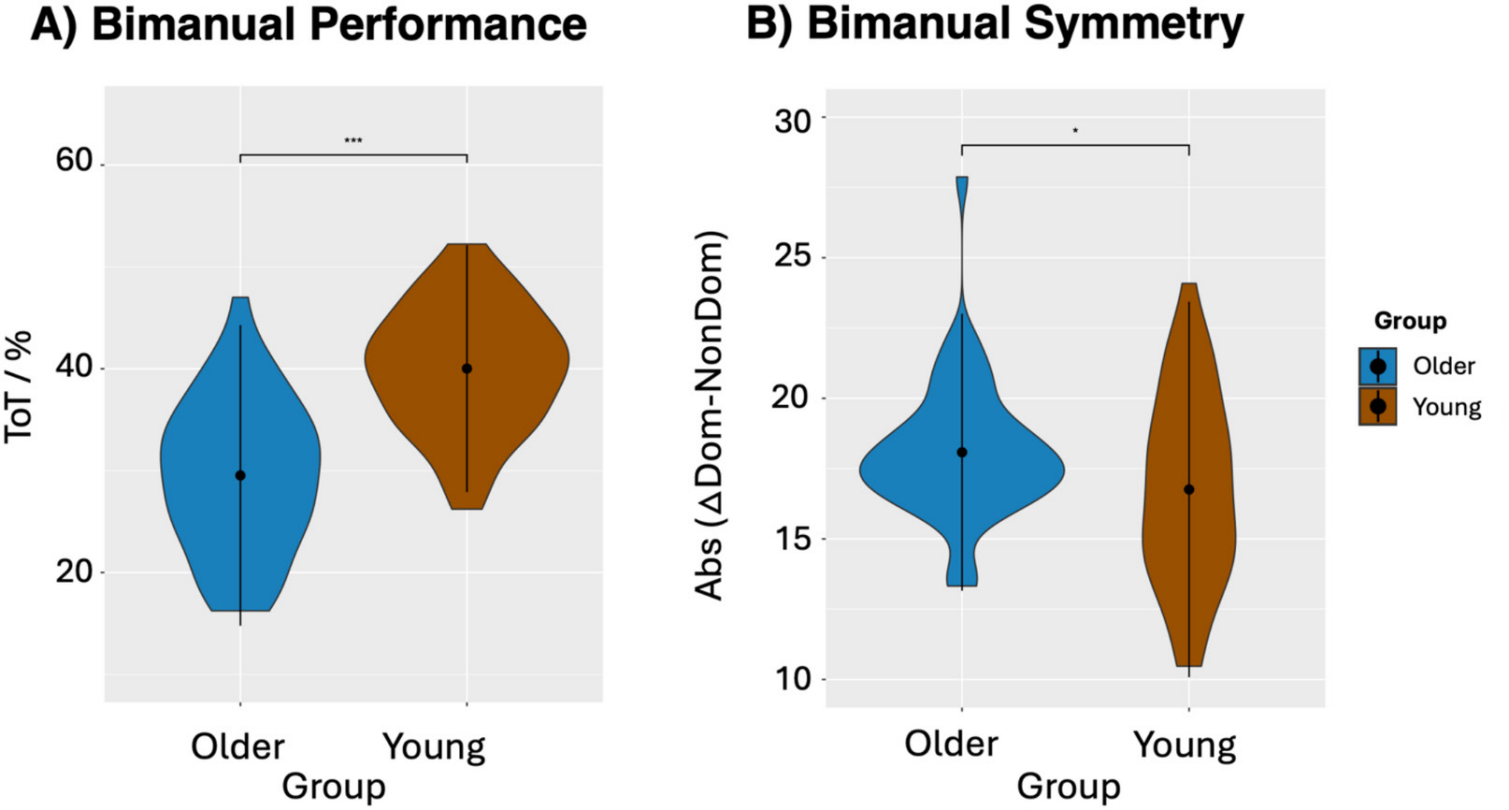
Time on target in the bimanual motor task for older and younger adults. (A) average time on target (ToT) of both hands during a trial as a measure of task performance. (B) Absolute difference in ToT in the dominant (Dom) and nondominant (NonDom) hand as a measure of bimanual symmetry. Results for older adults are shown in blue, results for younger adults are shown in red. Stars denote significance values: *p < 0.05, **p < 0.01, ***p < 0.001.

Performance during the dynamically changing task was measured by the fraction of time spent on target (ToT). Both ToT and absolute hand difference in ToT were normally distributed (Shapiro–Wilks test, *p* > 0.05). The young age group showed significantly higher ToT values compared with older adults (M_YA_ = 40.0%, M_OA_ = 29.5%, *p* < 0.001; Figure 2). The older age group showed a significantly larger absolute difference between the left and right hand (M_OA_ = 18.1%, M_YA_ = 16.8%, *p* = 0.0333; Figure 2B). ToT correlated with manual dexterity as measured by the Purdue Pegboard Assembly task when combining both groups (Spearman's rho = 0.354, *p* < 0.001). However, this relationship was largely attributable to between‐group differences. There was no significant correlation within either the young (Spearman's rho = −0.055, *p* = 0.716) or older (Spearman's rho = −0.085, *p* = 0.575) age group.

### 3.2 Group Differences in FA and Cortical Thickness

**FIGURE 3:**
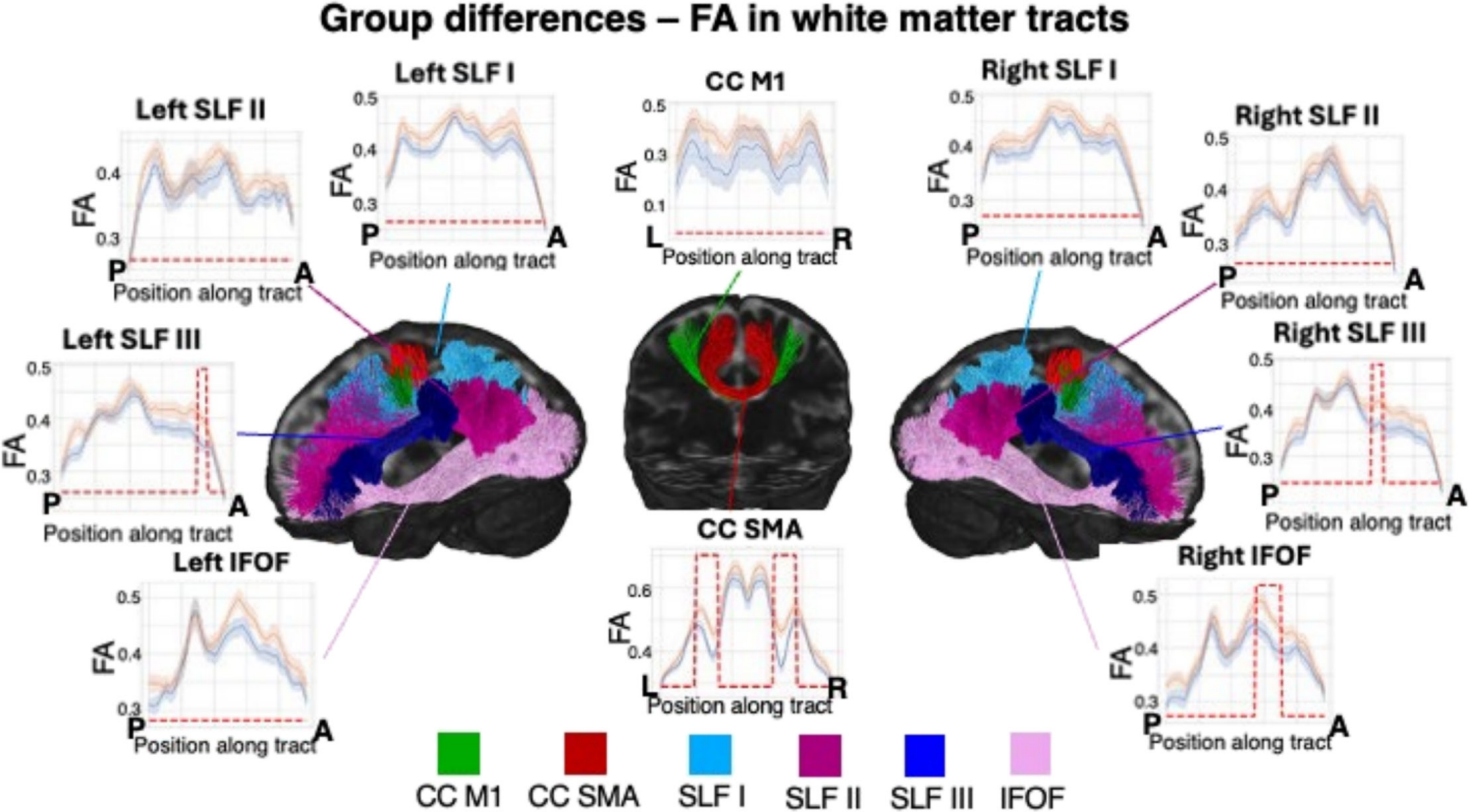
Group differences in fractional anisotropy (FA) in the tracts connecting the bimanual control nodes. FA values of older age group are displayed in blue while FA values of the younger age group are shown in red. Position labels on the *x*‐axis indicate left (L), right (R), anterior (A), and posterior (P). The dotted red lines indicate at which tract locations the groups show significant differences (corrected for multiple comparisons). Tracts are color‐coded: corpus callosum (CC; green), superior longitudinal fasciculus I (SLF I; light blue), superior longitudinal fasciculus II (SLF II; purple), superior longitudinal fasciculus III (SLF III; dark blue) and inferior fronto‐occipital fasciculus (IFOF; pink).

The younger age group had greater FA in segments of the CC SMA, right IFOF, and the bilateral SLF III when compared with the older adults (Figure 3). No other tracts showed a significant group difference. Mean diffusivity was significantly lower for the younger compared with the older age group in the bilateral IFOF and right SLF I (see Figure S2).

**FIGURE 4:**
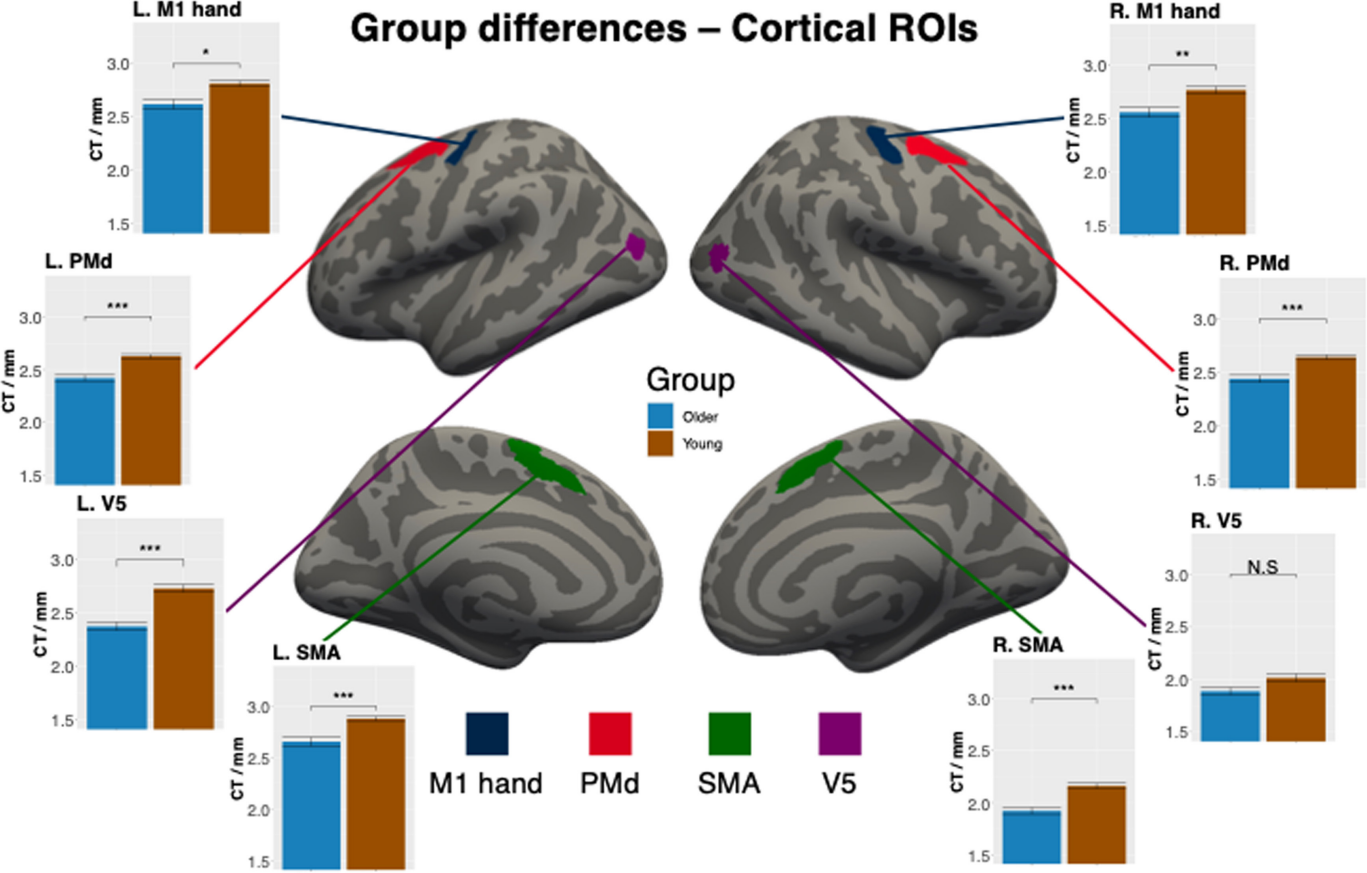
Cortical regions of interest (ROIs) within the bimanual control network. ROIs comprise the hand know of the primary motor cortex (M1 hand; black), dorsal premotor cortex (PMd; red), supplementary motor areas (SMA; green), and middle temporal visual areas (V5; purple). The younger age group (red bars) had a significantly higher cortical thickness (CT) than the older (blue bars) in all ROIs except for the right V5 area. Significance markers: *p < 0.05, **p < 0.01, ***p < 0.001, Bonferroni corrected between ROIs. L and R denote left and right hemispheres.

Concerning cortical morphometry, ChT was higher in younger than older age group across large stretches of the cerebral cortex (see Figure S3). When limiting the areas to the bimanual ROIs, all but the right V5 showed significantly higher CTh in the younger than older age group (Figure 4).

### 3.3 Structural Correlates of Dynamic Bimanual Task Performance

**FIGURE 5:**
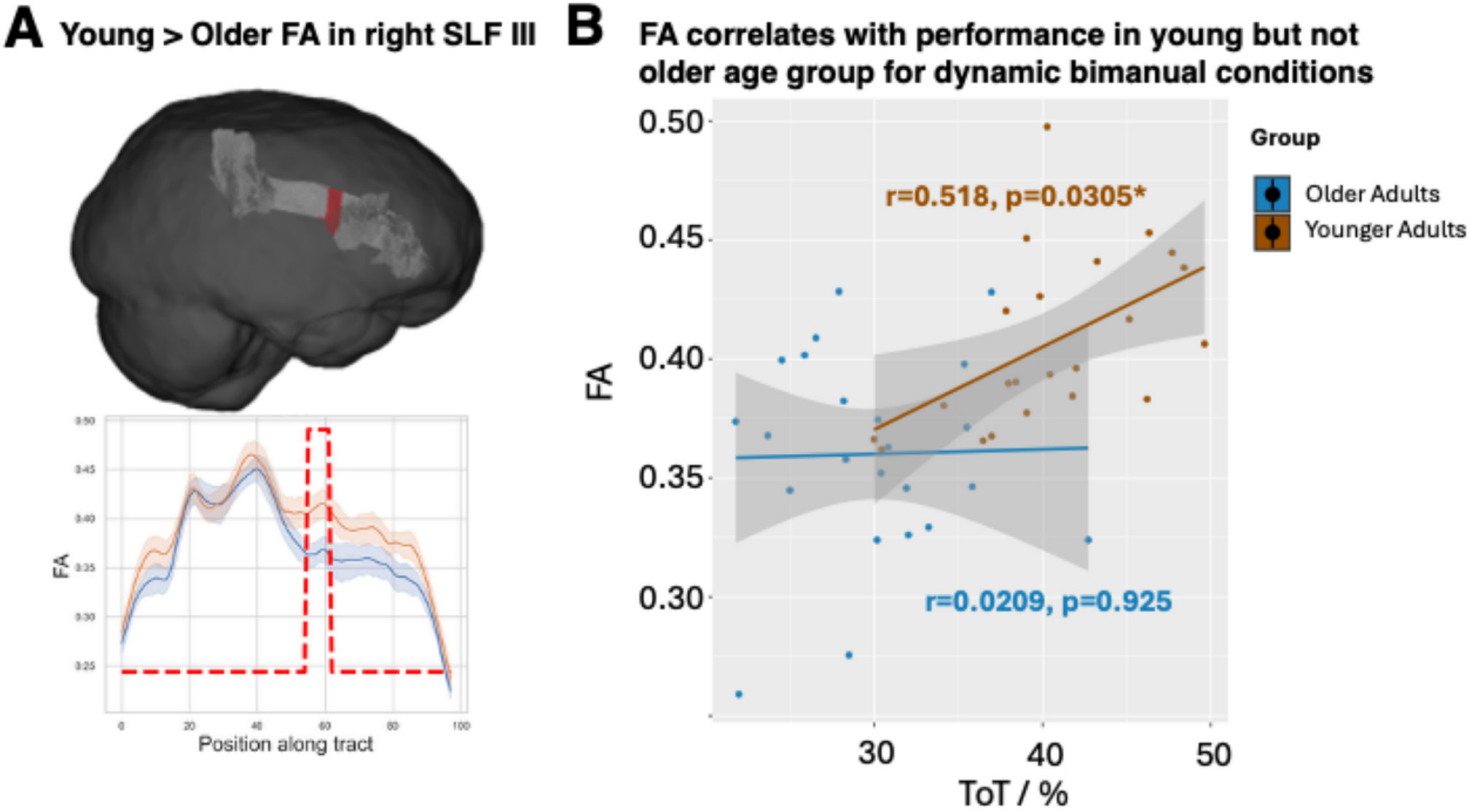
(A) Significant group differences in an anterior cluster of the right superior longitudinal fasciculus part III (SLF III). (B) Correlations between fractional anisotropy (FA) and performance (time on target; ToT) within the cluster showing significant age‐dependent effects.

One of the white tract segments showing age‐dependent FA decreases also exhibited a significant correlation with ToT. In the right SLFIII, higher ToT correlated positively with FA in the young but not in the older age group after correcting for multiple comparisons (YA: *r* = 0.51; *p* = 0.0198; OA *r* = 0.02; *p* > 0.9) (Figure 5), indicating that higher FA values were related to better performance in the younger age group only. However, the difference in correlation between age groups was not statistically significant (*z* = 1.73, *p* = 0.0845). Cook's distance was lower than 1 for all data points, indicating the absence of influential outliers. To make sure that the segment identified by group‐dependent differences was representative of the structure–performance of the entire tract, we explored the correlation statistics for the entire tract in both younger and older adults. This analysis confirmed that significant positive structure performance relations between the right SLF and ToT could only be detected in the younger age group, and *r* values were generally higher in the younger compared with the older age group (YA: mean *r* value. 0.193 ± 0.170; OA: mean *r* value −0.074 ± 0.142; see Figure S4). To investigate the lateralization of the structure–performance correlations to the right SLF III, we also calculated correlations for left hemisphere segments of SLF III. These results indicate that the correlations with task performance are specific to the right SLFIII (YA: *r* = −0.096, *p* > 0.6; OA: *r* = −0.266, *p* > 0.8). Correlations between FA and ToT in all significant group difference segments are reported in Table 2. To explore whether high versus low performers within each age group differ in their brain–behavior associations, we additionally analyzed high‐ and low‐performing subgroups separately, but found no significant differences in the strength of the FA–performance relationship (S5).

**TABLE 2:** Pearson's *r* values and uncorrected *p* values for correlations between FA and ToT in all segments showing significant group differences. *R* values labeled in bold survived multiple comparison correction.

FA	CC SMA 1	CC SMA 2	R. IFOF	R. SLF III	L. SLF III
Older ToT	*r* = −0.198	*r* = −0.197	*r* = 0.061	*r* = 0.024	*r* = −0.266
	*p* = 0.818	*p* = 0.816	*p* = 0.391	*p* = 0.457	*p* = 0.891
	*R* ^2^ = 0.039	*R* ^2^ = 0.039	*R* ^2^ = 0.004	*R* ^2^ = 0.001	*R* ^2^ = 0.071
Young ToT	*r* = −0.148	*r* = 0.284	*r* = −0.092	*r* = 0.519*	*r* = −0.096
	*p* = 0.745	*p* = 0.100	*p* = 0.659	***p* = 0.006**	*p* = 0.664
	*R* ^2^ = 0.022	*R* ^2^ = 0.081	*R* ^2^ = 0.008	***R*** ^**2**^ **= 0.269**	*R* ^2^ = 0.009

There were no correlations between ToT and MD in any of the significant segments for either of the age groups (*p* > 0.15). For CT, there were also no significant correlations with ToT in any of the ROIs (*p* > 0.08). Correlations between average CTh in ROIs and ToT are reported in Table 3.

**TABLE 3:** Correlation coefficients and uncorrected *p* values for correlations between CTh and ToT in all ROIs. Pearson's *r* for all except for L. M1 hand ROI (Spearman's rho). No correlation was significant after Bonferroni correction across the eight ROIs.

CT	L. M1 hand	L. PMd	L. SMA	L. V5	R. M1 hand	R. PMd	R. SMA	R. V5
Older ToT	rho = 0.327	*r* = 0.068	*r* = −0.185	*r* = −0.073	*r* = 0.162	*r* = −0.038	*r* = 0.011	*r* = −0.177
	*p* = 0.074	*p* = 0.385	*p* = 0.211	*p* = 0.377	*p* = 0.242	*p* = 0.436	*p* = 0.482	*p* = 0.222
	rho^2^ = 0.107	*R* ^2^ = 0.005	*R* ^2^ = 0.034	*R* ^2^ = 0.005	*R* ^2^ = 0.026	*R* ^2^ = 0.001	*R* ^2^ < 0.001	*R* ^2^ = 0.031
Young ToT	rho = −0.081	*r* = 0.197	*r* = −0.360	*r* = −0.211	*r* = 0.392	*r* = 0.238	*r* = 0.002	*r* = 0.206
	*p* = 0.364	*p* = 0.197	*p* = 0.0545	*p* = 0.179	*p* = 0.040	*p* = 0.149	*p* = 0.496	*p* = 0.185
	rho^2^ = 0.007	*R* ^2^ = 0.039	*R* ^2^ = 0.130	*R* ^2^ = 0.045	*R* ^2^ = 0.154	*R* ^2^ = 0.057	*R* ^2^ < 0.001	*R* ^2^ = 0.042

### 3.4 Hemispheric Asymmetries Correlate With Intermanual Symmetry

**FIGURE 6:**
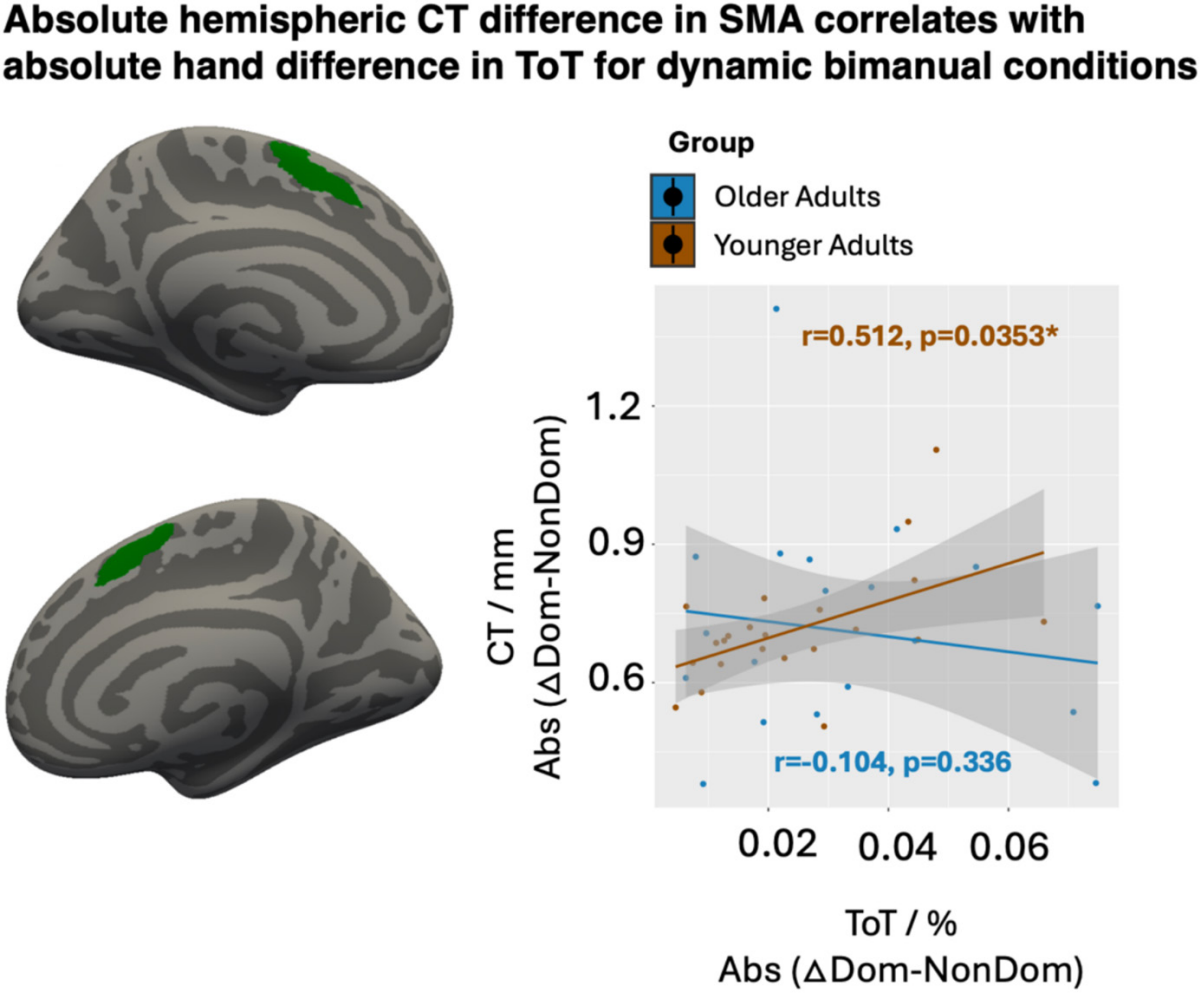
Absolute Time on Target difference between the hands correlates with absolute hemispheric difference in cortical thickness in the SMA in the young but not the old group. On the left, the spatial location of the cluster is visualized. On the right, the correlation between hemispheric symmetry in cortical thickness (CT) (defined as the difference in CT between the dominant [Dom] and nondominant [NonDom] hemisphere) and hemispheric symmetry in bimanual performance (defined as the difference in time on target [Tot] between the Dom and NonDom).

There was a significant correlation between absolute hand difference in ToT and the absolute hemispheric difference in CTh in the SMA in the young but not in the older age group (Figure 6) after correcting for multiple comparisons. Correlations for all cortical ROIs are reported in Table 4. Cook's distance was lower than 1 for all datapoints, indicating absence of influential outliers. However, the difference in correlation between age groups was not statistically significant (*z* = 1.21, *p* = 0.227). There were no significant correlations between absolute hand difference and the absolute difference in average FA or MD between the left and right hemispheric homolog tracts (all *p* values > 0.2). Furthermore, there was no significant correlation between mean FA or MD of the CC SMA and neither absolute hemispheric difference in CTh of the SMA, nor the absolute hand difference in ToT (S6).

**TABLE 4:** Correlation values and uncorrected *p* values for all hand and hemisphere difference values for each age group. Spearman's rho for all except for V5 ROI (Pearson's *r*).

Abs (△Dom − NonDom) CTh	M1 hand	PMd	SMA	V5
Older abs (△Dom − NonDom) ToT	*r* = −0.203	*r* = −0.200	*r* = −0.104	*r* = 0.376
	*p* = 0.202	*p* = 0.206	*p* = 0.336	*p* = 0.056
	*R* ^2^ = 0.041	*R* ^2^ = 0.040	*R* ^2^ = 0.011	*R* ^2^ = 0.141
Young abs (△Dom − NonDom) ToT	*r* = −0.047	*r* = −0.408	*r* = 0.512*	*r* = −0.378
	*p* = 0.42	*p* = 0.033	***p* = 0.009**	*p* = 0.045
	*R* ^2^ = 0.002	*R* ^2^ = 0.166	***R*** ^**2**^ **= 0.262**	*R* ^2^ = 0.143

## 4 Discussion

This study investigated how bimanual motor control, white matter microstructure, and cortical thickness within a predefined bimanual motor control network differ between healthy, right‐handed, younger and older adults. In addition, it also explored whether structures that showed significant age group differences in tissue quality correlated more strongly with bimanual performance in the older age group compared with the younger. Our findings corroborate previous literature, showing significantly lower tissue quality in the older group (Kang and Cauraugh 2022; Krehbiel et al. 2017; Leiberg et al. 2025; Roman‐Liu and Tokarski 2021; Rudisch et al. 2020; Vieluf et al. 2015; Yeatman et al. 2014). The younger group had significantly greater FA values in tracts related to visuomotor processing and bimanual coordination, specifically in extended sections of the anterior bilateral IFOF and SLF III as well as in bilateral mediolateral CC connecting the left and right SMA. The younger group also showed significantly higher cortical thickness in an extended cluster spanning predominantly frontal, parietal, and temporal regions. Within a bimanual motor network, we found that cortical thickness was significantly lower in the older age group in all but the right V5 area, that is, the bilateral M1 hand, PMd, SMA, and left V5. These structural differences were accompanied by decreased bimanual performance (lower ToT) and interhand performance symmetry in the older age group.

An exploratory analysis of the relationship between tissue quality and bimanual motor control in regions with significant group differences did not find evidence of stronger structure–performance correlations in the older age group. On the contrary, significant correlations were only found in the group of younger adults across behavioral outcomes, and our findings especially highlight the integrity of the right SLF III as functionally relevant in dynamic adaptations in a visually guided bimanual force task. While the correlations were not significantly different in slope when directly comparing the two groups, they may still highlight the functional relevance of intra‐hemispheric tracts, particularly the SLF III, for bimanual motor control and offer a basis for future investigation of the functional role of intrahemispheric tracts, leading bimanual research beyond the traditionally emphasized corpus callosum (Fling et al. 2011; Serbruyns et al. 2015).

### 4.1 Age Group Differences in the Bimanual Control Network

Our findings show a significant and bilateral lower FA in the older compared with the younger age group in the anterior portion of two major association tracts related to visuomotor integration, namely the SLF III and the IFOF. Both SLF III and IFOF are major association tracts related to visuomotor integration, and previous studies have indicated that the volume of these tracts shows positive correlations with good visuomotor control in both younger and older adults (Budisavljevic et al. 2017; Rogojin et al. 2023). In addition, our findings also show significant group differences in interhemispheric tracts. In bilateral segments of the callosal tract connecting the bilateral SMA, older adults showed significantly lower FA values compared with younger adults, while FA values in callosal tracts connecting the bilateral M1 did not show significant group differences. This finding is in line with previous evidence indicating that anterior premotor sections of the corpus callosum are more affected by aging than more posterior segments (Ota et al. 2006; Sullivan et al. 2006), potentially suggesting a preferential vulnerability of higher‐order motor integration pathways over primary motor execution pathways. We also found evidence for a widespread, age‐related decrease in cortical thickness, with frontal and heteromodal associative regions showing the earliest signs of cortical thinning, which is well aligned with previous literature (McGinnis et al. 2011; Salat et al. 2004). When focusing on the predefined bilateral motor network, we saw significantly decreased cortical thickness in all ROIs except for the right V5, demonstrating that a significant age‐related decrease in cortical thinning is not restricted to frontal and heteromodal regions.

### 4.2 Age‐Group Differences in the Relationship Between White Matter Microstructure and Bimanual Performance

We did not find evidence for stronger structure–performance relationships in healthy older adults compared with younger adults within the regions examined. Instead, we identified a cluster in the mid‐anterior portion of the right SLF III that was associated with performance only in the young adults; however, the difference in correlations between age groups was not statistically significant. No significant relationship was observed between bimanual coordination and callosal integrity. This contrasts with previous literature, where associations between white matter integrity within the corpus callosum and bimanual performance have been reported either across age groups (Zivari Adab et al. 2020) or specifically in older adults (Fling et al. 2011; Serbruyns et al. 2015; Fujiyama et al. 2016). By comparison, evidence from nonmotor domains aligns more closely with the present pattern of stronger structure–performance coupling in younger adults. For instance, a structural equation modelling study spanning ages 18–88 demonstrated that covariance between intrahemispheric association tract integrity and memory performance declines with age, indicating stronger coupling in younger individuals (de Mooij et al. 2018). Taken together, these observations raise the possibility that intrahemispheric association tracts may follow a structure–performance trajectory, showing reduced coupling with age. This interpretation is consistent with functional connectivity findings indicating increased interhemispheric coupling alongside decreased frontoparietal intrahemispheric connectivity in older adults (Chen et al. 2019). Consequently, a predominant focus on the corpus callosum may obscure age‐related changes occurring within intrahemispheric networks. However, given that the present study did not observe statistically significant differences between age groups, this interpretation should be considered preliminary and requires replication in larger samples.

### 4.3 Anterior Cluster in Right SLF III Correlates With Dynamic Bimanual Task Performance in the Young Group

Higher FA in a mid‐anterior cluster in the right SLF III correlated positively with performance in the dynamic bimanual conditions in the young adults. Structurally, the SLF III connects the inferior parietal cortex with the inferior frontal cortex (Janelle et al. 2022). Functionally, its role is lateralized, meaning that the left SLF III is mostly implicated in speech production (Kargar and Jalilian 2024; Maldonado et al. 2011), while the right SLF III has a crucial role in regulating attention and awareness in visuomanual tasks, such as reaching, grasping, and tool use (Gooijers and Swinnen 2014; Lazari et al. 2021; Ramayya et al. 2010; Seer et al. 2022; Wang et al. 2016) which fits with our findings highlighting the relevance of the right SLF III in a visually guided pinch‐grip task. Studies have shown that the volume of the right SLF II and III correlates with faster visuomotor processing (Budisavljevic et al. 2017). The correlation between tract microstructure and performance was only found for FA. MD and FA quantify the rate of diffusion averaged over all measured directions and how restricted the diffusion is to the dominating direction, respectively. FA is thus positively associated with axonal myelination, nerve fiber alignment and degree of orientation, while MD is related to cell density (as it reduces freely moving extracellular water), cellular swelling, and less myelin (Beaulieu 2014; Brabec et al. 2023; Chang et al. 2017). Hence, performance correlated with a metric sensitive to how well formed and homogenous the tract is (e.g., without influence of crossing fibers). We did not find any significant correlations between CTh in the predefined ROIs of the bimanual motor network and bimanual performance as measured by ToT.

### 4.4 Performance Asymmetry Between Hands Correlates With Hemispheric Asymmetry in Cortical Thickness of SMA in the Younger Group

While our main performance measure was a composite score of the left and right hands, due to a strong correlation between ToT measures in both hands, we were also interested in exploring if intermanual symmetry in performance correlated with hemispheric asymmetries in white matter microstructure and cortical thickness. We found that, for the younger age group, bimanual symmetry correlated with the absolute difference in cortical thickness in the SMA. This finding agrees with previous fMRI findings from our group in suggesting a crucial role of the SMA in maintaining bimanual symmetry. In the previous work, the blood oxygenation level–dependent (BOLD) signal was recorded in a group of young volunteers during the same task, and the study found that SMA activity, indexed by the BOLD signal, was also predictive of bimanual symmetry during the bimanual pinch‐force task (Karabanov et al. 2023). Together, these two studies show that both interhemispheric differences in CTh and functional activity of the SMA are critical for synchronous bimanual control, highlighting the importance of this region in coordinating motor output across both hands. It is, however, important to point out that the interhemispheric difference in CTh of the SMA did not correlate with the tissue microstructure parameters of the CC SMA, indicating that the difference in CTh between hemispheres is not explained by differences in structural connectivity. Hemispheric asymmetries in CTh could instead be due to congenital differences or experience‐driven plasticity, highlighting the need for longitudinal brain developmental and bimanual motor training intervention studies.

### 4.5 Study Limitations and Future Directions

This study is exploratory, focuses on secondary outcome measures, and is based on a relatively small, homogenous sample, limiting statistical power and generalizability. Older adults typically exhibit greater interindividual variability in both brain structure (Ziegler et al. 2012) and behavior (Hofer et al. 2002), and the present findings therefore require replication in larger, confirmatory studies. Importantly, the absence of a significant structure–function association in the older group should not be interpreted as evidence of absence; given the increased interindividual variability in older adults, this study may have been underpowered to detect such associations. In addition, the study population was relatively homogeneous (right‐handed, well‐educated Danish adults with moderate‐to‐high physical fitness), which further limits generalizability and may attenuate observable age‐related differences. Furthermore, the cross‐sectional design precludes causal inference about aging effects and is susceptible to cohort and survivorship biases (Hofer et al. 2002). Future studies should therefore include larger and more diverse samples, longitudinal designs, and more direct assessments of physical fitness and cognitive function to better characterize their contributions to brain structure and performance across aging.

We believe that a key strength of the present work is its emphasis on the relevance of tracts beyond the corpus callosum in associations with (bi)manual motor control. Identifying prospective target tracts like the SLF III is important because studies investigating brain–behavior relationships often limit their analyses to a predefined set of anatomical structures, potentially overlooking critical pathways. This is necessary because brain‐wide association studies require very large data sets (*N* > 100) (Marek et al. 2022). In a resource‐intensive technique like neuroimaging, such numbers are often prohibitively expensive and time‐consuming, and exploratory work is needed to justify targeted analyses in smaller samples. Moreover, this study was cross‐sectional, and more longitudinal studies are needed to make stronger inferences about directionality and potential causality of brain–behavior relationships (Dohm‐Hansen et al. 2024).

## 5 Conclusions

In this study, we investigated differences in white matter structure and cortical thickness, focusing on a cortical bimanual motor control network, between groups of young and older healthy adults. In regions where age group differences could be detected, we examined whether there was an age group difference in structure–performance coupling. We found that a segment in the anterior SLF III was associated with better bimanual performance in younger adults, but we did not find evidence for our initial hypothesis that structure–performance coupling would be increased in older adults. Our findings highlight that fiber tracts beyond the corpus callosum can show significant structure–performance associations. Future studies should investigate whether there are systematic differences between interhemispheric callosal tracts—where increasing associations with age are more consistently reported—and intrahemispheric association tracts, for which our data may offer tentative evidence of decreasing structure–function coupling in older adults.

## Author Contributions

**Mikael Novén:** conceptualization, investigation, writing – original draft, visualization, software, formal analysis, data curation. **Jesper Lundbye‐Jensen:** conceptualization, writing – review and editing. **Henrik Lundell:** methodology, supervision, writing – review and editing. **Hartwig Roman Siebner:** conceptualization, writing – review and editing, resources. **Anke Ninija Karabanov:** conceptualization, funding acquisition, writing – original draft, supervision, project administration.

## Funding

This work was supported by Danmarks Frie Forskningsfond (Grant number 0169‐00027B; Grant holder AK). H.L. is supported by the European Research Council (ERC) under the European Union's Horizon 2020 research and innovation programme (grant agreement No 804746).

## Ethics Statement

The studies involving human participants were reviewed and approved by Research Ethics Committees of the Capital Region (De Videnskabsetiske Komitéer‐Region H; Journal‐nr: H‐21025010).

## Consent

The participants provided their written informed consent to participate in this study.

## Conflicts of Interest

The authors declare no conflicts of interest.

## Supporting information


**Data S1:** Supporting Information

## Data Availability

The data supporting the conclusions of this article will be made available by the authors, without undue reservation.
