# Supplementary material for: Anterior Superior Longitudinal Fasciculus Microstructure Correlates With Bimanual Visuomotor Performance in Healthy Younger but Not Older Adults
Source: Eur J Neurosci. 2026 Jul 14;64(2):e70619. doi: 10.1111/ejn.70619 (PMC13369776; doi:10.1111/ejn.70619)
Supplement: Supplementary file 1 — Data S1: Supporting Information [file EJN-64-0-s001.docx]

# **S1 Correlations in performance between hands for dynamically alternating task conditions**

Time on Target (ToT) correlates between hands in the dynamic task conditions. Plot are shown in Figure S1. Performance measures from the dynamically alternating conditions correlated between hands for each group (yellow: younger // blue: older).


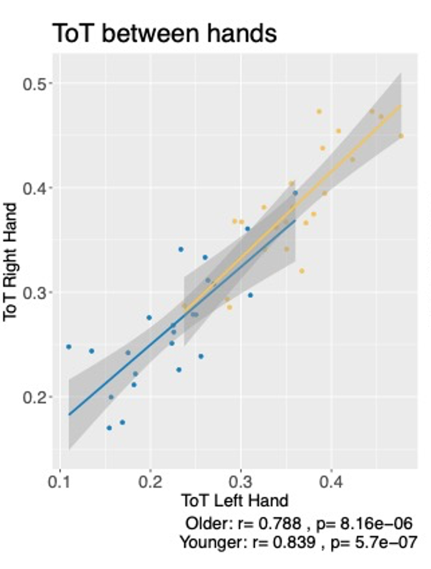


# **S2: Group differences in tracts of interest in MD**

Mean diffusivity differed significantly between groups in multiple segments in the tracts of interest. Segments showing significant group differences marked by the red square line.


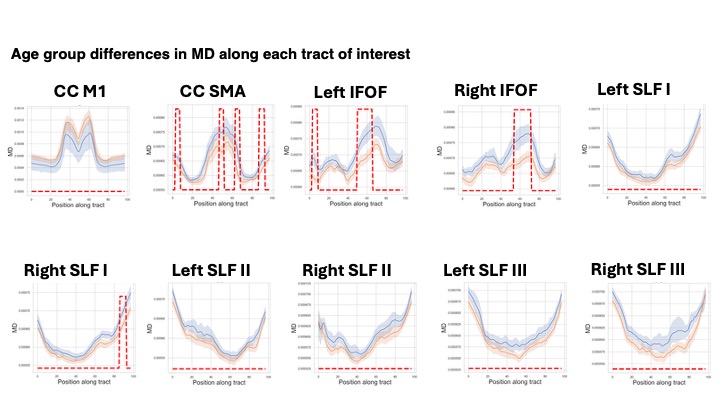


# **S3:Group differences in cortical thickness**

The older group had significantly (10000 permutations, p threshold: 0.001, cluster-wise p threshold: 0.05) lower cortical thickness in widespread cortical regions. Positive (heat) means areas where the older have a thinner cortex compared to the younger. Color bar indicates –log(p).


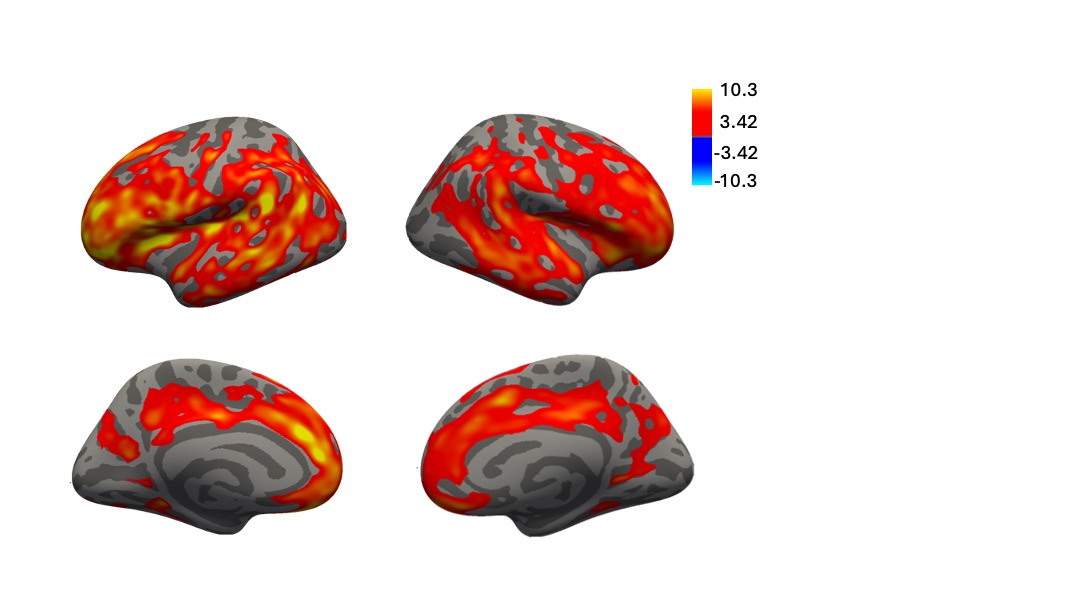


# **S4:Correlation statistics along right SLF III**

To supplement the finding that Time on Target with FA in an anterior segment of the right SLF III in the young group, Pearson’s r for correlations between Right SLFIII FA and Time on Target for the young and the old group. Each bar shows the Pearson’s r for a segment of the tract. Black are segments showing significant (p<0.05) correlations
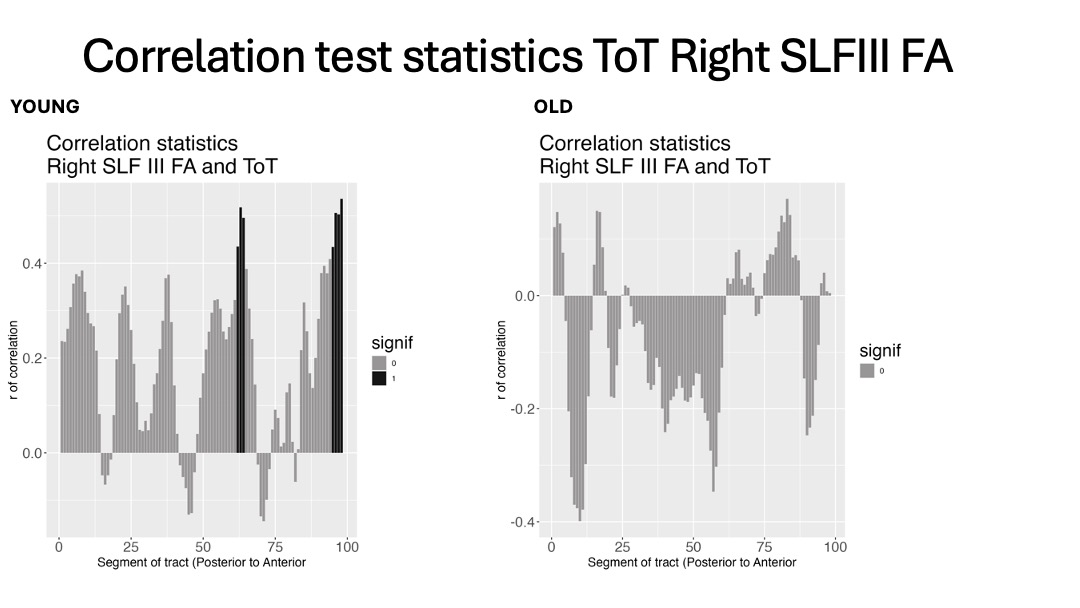


# **S5: Right SLF III anterior segment correlations in high vs. low performers in each age group**

To explore potential differences in FA-ToT correlations in the right anterior SLF III segment between high and low performers in each age group, the correlation analysis was also performed on the top and bottom half performers (median split) within each age group. This analysis showed that there was no correlation for either level of performers in the older age group (high: r=0.109, R^2^=0.0119, p=0.368, n=12; low: r=0.203, R^2=0.0412, p=0.275, n=11) and neither in the high-performing young group (r=-0.017, R^2^<0.001, p=0.520, n=11). However, there was a significant correlation in the low-performing young group (r=0.620, R^2^=0.384, p=0.021, n=11). None of the performance subgroups within the age groups differed significantly in their brain-performance correlations (old high vs. low performers: p=0.771; young high vs. low performers: p=0.199).

**S6: SMA-SMA CC tracts correlations with Abs (△Dom-NonDom) CTh and abs (△Dom-NonDom) ToT**

There were no correlations between average FA or MD along the SMA-SMA CC tract with either absolute hemispheric difference in cortical thickness of the SMA or with absolute hand difference in task performance (ToT) in any of the age groups.

**Abs (△Dom-NonDom) CTh**

| **Age group** | **Mean FA in SMA-SMA CC** | **Mean MD in SMA-SMA CC** |
| --- | --- | --- |
| Young | r=-0.002, p=0.995 | r=-0.024, p=0.916 |
| Older | r=-0.182, p=0.430 | r=0.164, p=0.465 |

**Abs (△Dom-NonDom) ToT**

| **Age group** | **Mean FA in SMA-SMA CC** | **Mean MD in SMA-SMA CC** |
| --- | --- | --- |
| Young | r=0.119, p=0.597 | r=-0.039, p=0.864 |
| Older | r=0.119, p=0.597 | r=0.305, p=0.167 |
